# Analyzing the psychological impact of the COVID-19 pandemic among turkish immigrants treated at the neuro-psychiatric center riem Munich

**DOI:** 10.1192/j.eurpsy.2021.1739

**Published:** 2021-08-13

**Authors:** E.D. Cindik Herbrüggen

**Affiliations:** 1 Psychosomatik/psychotherapie, Neuro-Psychiatric Center Riem (Neuro-Psychiatrisches Zentrum Riem), München, Germany; 2 Psychiatrie & Psychotherapie, Neuro-Psychiatric Center Riem (Neuro-Psychiatrisches Zentrum Riem), München, Germany

**Keywords:** COVID-19, Turkish immigrants, corona pandemic, immigrants’ mental health

## Abstract

**Introduction:**

Infectious diseases have been humanity’s constant problem throughout history and they have shown how vulnerable we remain. COVID-19, commonly known as coronavirus pandemic, has already triggered a variety of psychological problems including fear, anxiety, and suicidal attempts. It has been hypothesized that immigrants who lost their job and have lower income or lower education level are more worried and fearful.

**Objectives:**

This paper aims to investigate the psychological conditions and stress level of immigrants in the NPZR. Moreover, the parameters influencing stress levels of the immigrants were analyzed.

**Methods:**

Demographics, level of stress and current psychological conditions of participants were gathered and analyzed through a structured survey. Besides, in-person interviews were conducted to explore the responses of the participants to receive more and deeper information.

**Results:**

The sample consists of 110 responders (45.3 % males; 51.8 % females). The findings of the study illustrated that while there was no relationship between gender of the immigrants and the level of stress, participants with low income (27.5 %) and unemployed immigrants (24.2 %) felt more worried and anxious. As time passes, the anxiety level of the participants decreased by 49.1 % but 40.9 % of the participants still experience severe anxiety.

**Conclusions:**

The findings demonstrate that having low income or being unemployed as well as the cancellation of travel plans, are positively related with the depression level of the participants. The result of this paper show that more attention has to be given towards immigrants with low income as they are more vulnerable during the COVID-19 Pandemic.

**Disclosure:**

No significant relationships.

